# Genetic characteristics of soybean resistance to HG type 0 and HG type 1.2.3.5.7 of the cyst nematode analyzed by genome-wide association mapping

**DOI:** 10.1186/s12864-015-1800-1

**Published:** 2015-08-13

**Authors:** Yingpeng Han, Xue Zhao, Guanglu Cao, Yan Wang, Yinghui Li, Dongyuan Liu, Weili Teng, Zhiwu Zhang, Dongmei Li, Lijuan Qiu, Hongkun Zheng, Wenbin Li

**Affiliations:** Key Laboratory of Soybean Biology in Chinese Ministry of Education (key Laboratory of Soybean Biology and Breeding/Genetics of Chinese Agriculture Ministry), Northeast Agricultural University, Harbin, 150030 China; Institute of Crop Science, National Key Facility for Crop Gene Resources and Genetic Improvement (NFCRI) Chinese Academy of Agricultural Sciences, 100081 Beijing, China; Bioinformatics Division, Biomarker Technologies Corporation, 101300 Beijing, China

**Keywords:** *Heterodera glycines*, Genome-wide association study, SCN resistance, HG Types

## Abstract

**Background:**

Soybean cyst nematode (SCN, *Heterodera glycines* Ichinohe) is one of the most fatal pests of soybean (*Glycine max* (L.) Merr.) worldwide and causes huge loss of soybean yield each year. Multiple sources of resistance are urgently needed for effective management of SCN *via* the development of resistant cultivars. The aim of the present study was to investigate the genetic architecture of resistance to SCN HG Type 0 (race 3) and HG Type 1.2.3.5.7 (race 4) in landraces and released elite soybean cultivars mostly from China.

**Results:**

A total of 440 diverse soybean landraces and elite cultivars were screened for resistance to SCN HG Type 0 and HG Type 1.2.3.5.7. Exactly 131 new sources of SCN resistance were identified. Lines were genotyped by SNP markers detected by the Specific Locus Amplified Fragment Sequencing (SLAF-seq) approach. A total of 36,976 SNPs were identified with minor allele frequencies (MAF) > 4 % that were present in 97 % of all the genotypes. Genome-wide association mapping showed that a total of 19 association signals were significantly related to the resistance for the two HG Types. Of the 19 association signals, eight signals overlapped with reported QTL including *Rhg1* and *Rhg4* genes. Another eight were located in the linked regions encompassing known QTL. Three QTL were found that were not previously reported. The average value of female index (FI) of soybean accessions with resistant alleles was significantly lower than those with susceptible alleles for each peak SNP. Disease resistance proteins with leucine rich regions, cytochrome P450s, protein kinases, zinc finger domain proteins, RING domain proteins, MYB and WRKY transcription activation families were identified. Such proteins may participate in the resistant reaction to SCN and were frequently found in the tightly linked genomic regions of the peak SNPs.

**Conclusions:**

GWAS extended understanding of the genetic architecture of SCN resistance in multiple genetic backgrounds. Nineteen association signals were obtained for the resistance to the two Hg Types of SCN. The multiple beneficial alleles from resistant germplasm sources will be useful for the breeding of cultivars with improved resistance to SCN. Analysis of genes near association signals may facilitate the recognition of the causal gene(s) underlying SCN resistances.

**Electronic supplementary material:**

The online version of this article (doi:10.1186/s12864-015-1800-1) contains supplementary material, which is available to authorized users.

## Background

Soybean cyst nematode (SCN, *Heterodera glycines* Ichinohe) is one of the most destructive pests of soybean (*Glycine max* (L.) Merr.). SCN suppresses soybean yield, and causes an estimated seed yield loss of approximately $1.5 billion dollars per year only in United States [1]. *H. glycine* probably evolved either in China or Japan and had been spread to the New World [2].

An SCN race was first recognized in 1954, and a total of 16 races were reported based on all the possible combinations on four soybean differentials including Peking, Pickett, PI 88788 and PI 90763 [3]. Recently, the HG Type based on eight diffferential cultivars was introduced to more accurately represent the *H. glycine* population types found in soil instead of the previously described race [4]. *H. glycine* is now widely distributed in more than 15 countries, particularly in those areas where soybean is grown on a commercial scale, like USA and China [5]. Hg Type 0 or 7 (race 3) was mainly distributed in the south of 37°N latitude in the United states. And the Hg Type 1.2.5- (races 4 and 14) were predominant for soybean production areas in the north of 37° N latitude in the United states [6]. In China eight races of SCN predominate (races 1, 2, 3, 4, 5, 6, 9 and 14) [2]. Hg Type 0 was predominant in the northeastern provinces of China (north of 41° N latitude), and Hg Type 1,2,5- was one of the two predominant races in Huang-Huai valleys (between 32° N and 41° N latitude in China) [2, 7].

A limited control of this pest is achieved by different forms of rotations and the application of pesticides. However, breeding cultivars with resistance has been the most effective and economical way to control SCN. A number of soybean lines have resistance to SCN, but only a few of them have been used to breed commercial soybean [8]. Currently, most SCN-resistant cultivars in the north central United States were developed from a single source of resistance, PI88788. Only a few cultivars were derived from PI 548402 (Peking) and PI 437654 (*via* ‘CystX’ or ‘Hartwig’) [9]. Pathogen populations have the ability to mutate, recombine and/or drift quickly to new Hg Types that overcome plant resistances [10]. Therefore, using the soybean varieties with the genetic background of a few (but not many) resistant cultivars, like PI88788 and Peking, would lead to the changes of predominant races and the loss of resistance [11]. Hence, multiple sources of resistance are urgently needed for effective management of SCN in the world.

The knowledge of genetic architecture of SCN resistance is very important for breeding resistance varieties [11]. So far, genetic architecture analyses mainly depended on traditional quantitative trait locus (QTL) linkage mapping based on bi-parental populations. Once the desired QTL were mapped, molecular markers that were tightly linked to the QTL could be applied in marker-assisted breeding to improve and shorten the process for developing cultivars with resistance to SCN. Many QTL conferring resistance to SCN in soybean have been mapped on almost all chromosomes (except of Gm02; LG D1b) based on SSR markers [12–19]. Of them, two major QTL, *Rhg1* on Gm18 and *Rhg4* on Gm08 [12], were identified in many different resistance sources. Genes and their alleles underlying both loci were identified from PI88788 and cultivar Forrest, respectively [20, 21]. Fine mapping, sequencing and gene analysis of *Rhg1* and *Rhg4* were reported [19–23]. The receptor like kinase at *Rhg1-a* caused pleiotropic resistance to sudden death syndrome and soybean cyst nematode as a transgene by altering signaling responses [23, 24]. The study on *Rhg1-b* allele’s locus by Cook *et al.* [20] considered the SCN resistance of *Rhg1* in PI 88788 was conferred by three genes, and the copy number variation of these genes caused the phenotypic differences between susceptible and resistant varieties. The SCN resistance of *Rhg4* in Forrest was controlled by a serine hydroxymethyl transferase gene, that acted in cellular one-carbon metabolism [21]. The mutation of this gene could disturb the concentration of folate and lead to a nutritional deficiency detrimental to the life cycle of SCN [21]. Although few SCN resistance genes have been cloned based on the identified QTL, the large confidence genomic intervals of most QTL impaired the precise identification of causative genes.

Association mapping, which exploits historical recombination events at the population level, could supplement linkage mapping in the dissection of complex trait variation [25]. Genome-wide association (GWAS) mapping, a specific association mapping stage, is a powerful complementary strategy for an alternation of classical bi-parental linkage mapping to dissect complex traits by using the naturally occurring population [26]. GWAS mapping approach has been used with success in studies on important traits of model plants and crops, such as *Arabidopsis thaliana*, rice, maize, sorghum, barley [27–37]. Recently, GWAS mapping of seed quality related traits and disease resistance of soybean were reported. Hwang *et al.* [37] identified 40 single nucleotide polymorphisms (SNPs) in 17 different genomic regions significantly associated with seed protein content using a data set of 55,159 SNPs. A major seed protein QTL has been previously mapped to the same location and potential candidate genes have recently been identified in this region. Twenty five SNPs in 13 different genomic regions associated with seed oil content were also detected by GWAS. GWAS mapping of *Sclerotinia sclerotiorum* resistance in soybean with a genotyping-by-sequencing approach [30], have also been used to identify QTL for sudden death syndrome using soybean reference genome sequence and high throughput SNP assays. Both known QTL and new quantitative trait nucleotide (QTN) were detected and candidate genes were predicted in the above two studies [30, 32]. Therefore, GWAS mapping could detect beneficial alleles from excellent germplasm with different genetic background in one mapping panel and obtain the fine map of association genomic regions and candidate genes by using sufficient density of SNP markers. Whole genome sequencing (WGS) is the most straightforward method for GWAS; however, WGS is not still affordable for most researcher, especially for genotyping hundreds of samples. Different form WGS, reduced representation sequencing have many advantage such as reducing the genome complexity and lower cost. To date, many reduced representation sequencing have been developed such as restriction site–associated DNA (RAD) tag sequencing 2b-RAD [38], and specific-locus amplified fragment sequencing (SLAF-seq) [39]. With comparison with other reduced representation sequencing method, SLAF-seq have definite advantage such as higher genotyping accuracy, lower sequencing costs, higher efficient detect system, which was successfully applied in rice [40], sesame [41] and soybean [42, 43]. The objectives of the present study were; 1) to investigate the genetic architecture of soybean resistance to SCN Hg Type 0 and Hg Type 1.2.3.5.7 by GWAS using reduced genomic sequencing data from 440 diverse soybean germplasm mostly collected from China where SCN resistant germplasm was enriched; 2) to evaluate the beneficial alleles of each SNP marker associated with the resistance to the two tested Hg Types in order to provide valuable markers to breed soybean lines with SCN resistance; and 3) to analyze the genetic landscape of each association signal to identify genes that may participate in the interaction of SCN and soybean roots.

## Results

### SNP genotyping

The genotyped samples included a set of 440 soybean germpasm collected mostly in China, representing a broad diversity of genotypes (Additional file 1). The Genomic DNA of the 440 accessions were partially sequenced using specific-locus amplified fragment sequencing (SLAF-seq) approach by Illumina Genome Analyzer II [39]. The 347 million paired-end reads with 45 bp read length were mapped to the reference soybean genome (Wm82.a2.v1) and a mean of 57,418 high quality tags were identified from paired-end reads for each accession. A data set of 36,976 SNPs at Minor Allele Frequency (MAF) ≥ 4 % was generated from 57,418 high quality tags (Additional file 2). The rate of missing data was controlled at less than 10 % for each accession. The set of SNPs covered 20 chromosomes of soybean and the SNP numbers ranged from 1,165 (Gm12) to 2,914 (Gm18) with an average number of 1,848 for each soybean chromosome. Because the length of *G. max* genome was around 950 megabases [44], the resulting SNP density of one SNP per 27 Kbp was available in the present study. This sequencing-based SNP map would offer benefits for mapping studies for many other soybean traits.

### Linkage disequilibrium (LD) and population structure analysis

**Fig. 1:**
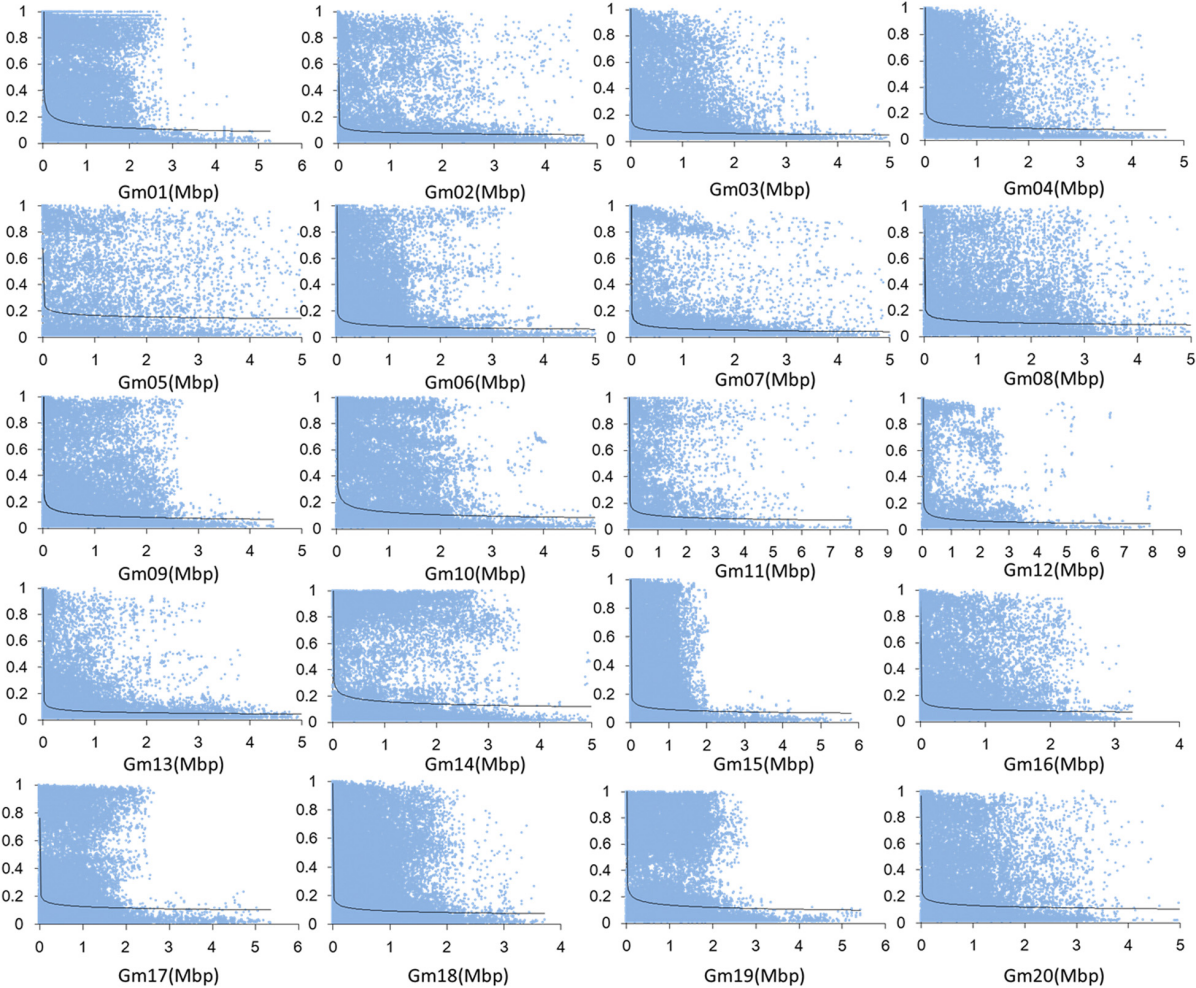
Linkage disequilibrium pattern of soybean genome

To characterize the mapping resolution for genome wide association study, the distributions of the average extent of LD decay and r^2^ between the different physical distances were quantified. LD decay for each chromosome was different (Fig. 1). The mean LD decay distance also varied among all chromosomes, ranging from 106 Kbp to 633 Kbp. The average r^2^ for all chromosomes was estimated at 227 Kbp. The marker density would meet the demand of GWAS mapping based on this LD pattern.

**Fig. 2:**
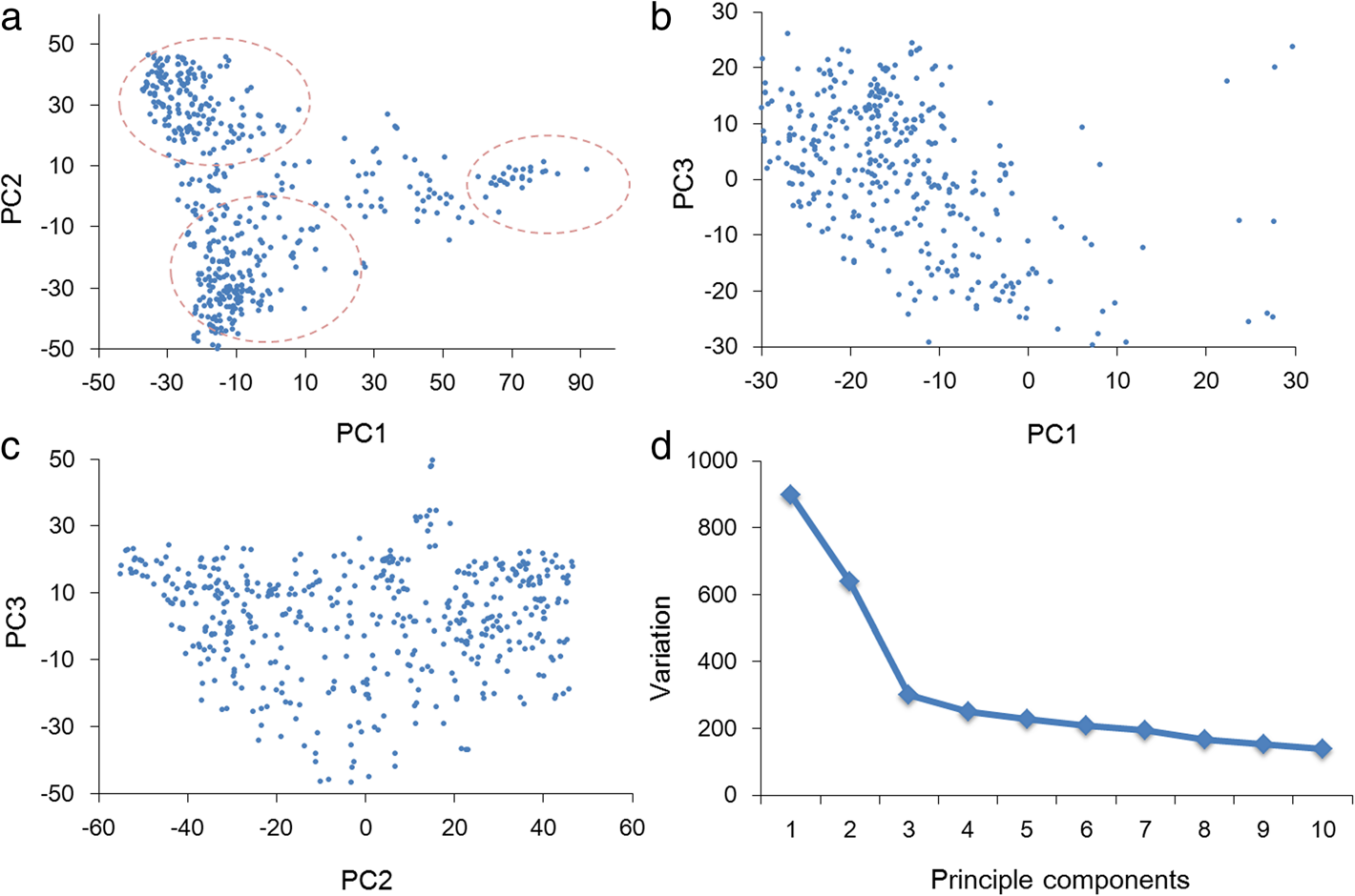
Population structure evaluation based on principle component analysis. **a**–**c** population structure was revealed by principle component 1 (PC1) and principle component 2 (PC2), PC1 and PC3, PC2 and PC3. **d** genetic variations of the first 10 PCs

To understand the population stratification of mapping panel, principle component analysis was conducted for the association panel based on 35,760 SNP markers. The result showed that the total amount of genetic variation explained by the first 10 principal components was only 24.4 %, indicating that the lines of the association panel were randomly sampled. The association panel was separated into three sub-populations by the first two PCs with some intermediate individuals among them (Fig. 2a). However, the accessions could not to be grouped clearly by either PC1 and PC3, or PC2 and PC3 (Fig. 2b–c). The genetic variation was decreased sharply at the point of PC3 (Fig. 2d). Therefore, the population structure could be well controlled with the first three PCs.

### Trait variation and genome-wide association study (GWAS)

**Fig. 3:**
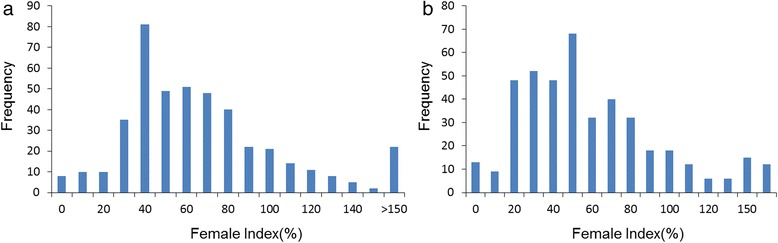
Distribution of Female Index (FI) among 440 soybean accessions. **a** FI distribution of HG Type 0 (SCN race 3). **b** FI distribution of HG Type 1.2.3.5.7 (SCN race 4)

The female index (FI) value of the phenotypic measurements of 440 soybean accessions for the GWAS was shown in Fig. 3. The observed FI values of soybean susceptibility to HG type 0 were 0–245.18 % with an average FI value of 53.14 %. FI values of HG type 1.2.3.5.7 resistant soybeans were 0–212.77 % with an average value of 58.23 %. The phenotypic data were in a condition of continuous distribution approximately.

**Fig. 4:**
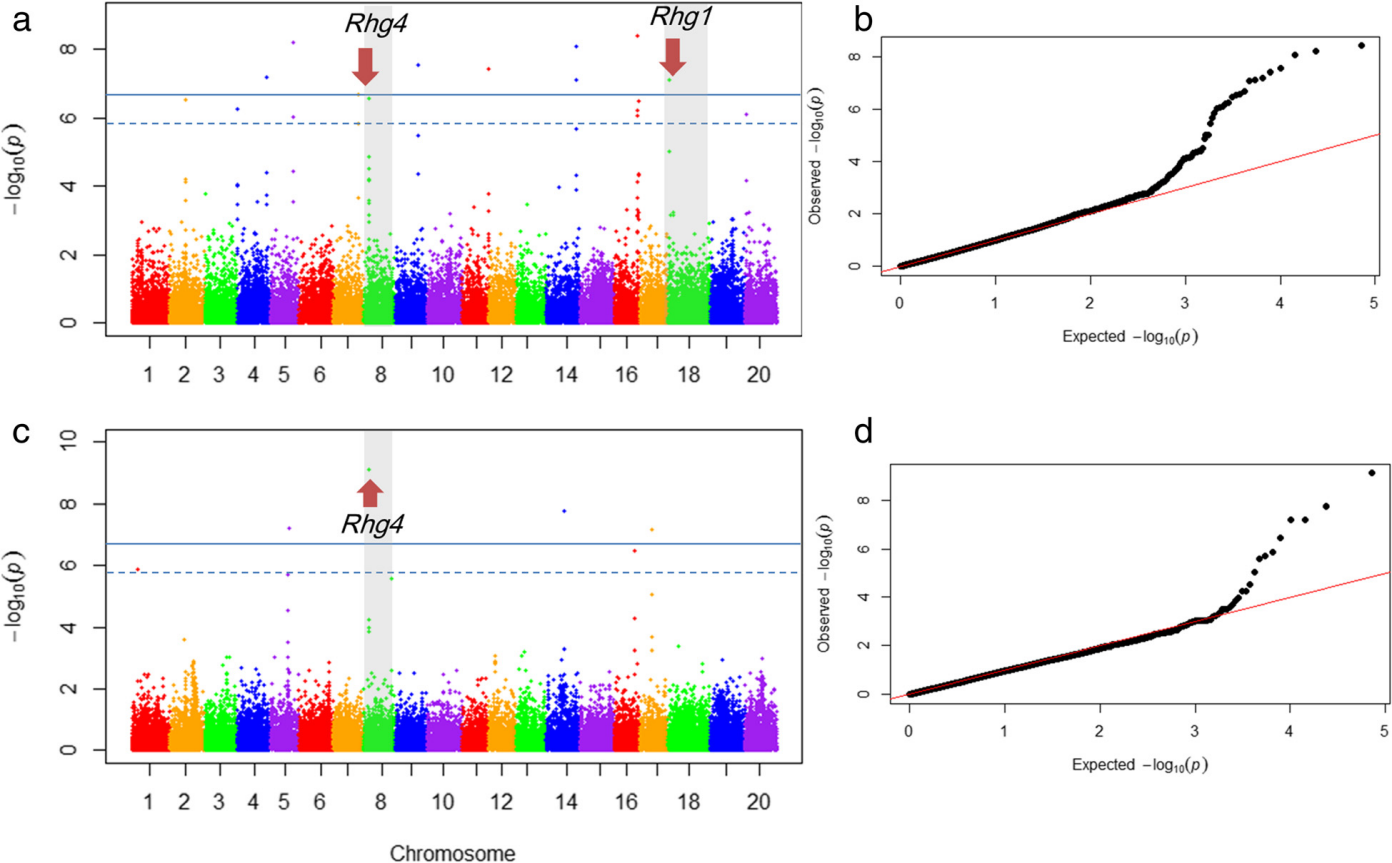
Genome wide association signals for soybean resistance to HG Type 0 and HG Type 1.2.3.5.7. **a**,**c** Genome wide Manhattan plots of associations from compressed mixed linear model. *x* axis showed the SNPs along each chromosome; *y* axis was the −log 10 (*P*-value) for the association for soybean resistance to Hg Type 0 and Hg Type 1.2.3.5.7, respectively. **b**,**d** Quantile-Quantile plots for mixed linear models of the resistance to Hg Type 0 and Hg Type 1.2.3.5.7

The genetic basis of natural variation for soybean resistance to SCN HG Type 0 and HG Type 1.2.3.5.7 were explored by using an association panel of 440 diverse germplasm genotyped with 36,976 SNP markers. A total of 19 association signals were identified with *P* < 1.35 × 10^−6^ for the two traits in a genome wide scan under compressed mixed linear model that controlled the population structure and familial relatedness (Fig. 4 and Table 1). Among the 19 association signals, eighteen were declared significant under the threshold of *P* < 2.70 × 10^−7^. The 19 peak SNPs explained 6.2–15.4 % of the total phenotypic variation. Twelve signals were significantly associated with the resistance to Hg Type 0 and another seven signals were found to be related to the resistance to Hg Type 1,2,3,5,7. The known SCN resistant loci, *Rhg1* and *Rhg4*, were identified by the present study for soybean resistance to Hg Type 0. The *Rhg4* locus was also found to contribute resistance to Hg Type 1.2.3.5.7. The rs33704130 located on Gm16 was significantly associated with the resistance for both Hg Types. It could be an important locus that offered resistance to multiple Hg Types. Eight signals overlapped with reported QTL, and another eight were located in the linked region of known QTL (Additional file 3). Three QTL were newly found in the present study.

**Table 1:** Peak SNP associated with resistance to the two Hg Types (race 3 and race 4) identified by GWAS and evaluation of beneficial alleles

SNP	Chromosome	Physical position	-log10(P)	Minor Allele Frequency	R^2^ of Model with SNP (%)	Resistant allele	Susceptible alleles	Average FI of accessions with resistant allele	Average FI of accessions with susceptible allele	Average FI of population	Hg Type^1^
rs8009636	Gm01	8009636	5.87	0.07	10.58	A	C	51.44	90.14	58.23	race 4
rs21804864	Gm02	21804864	6.54	0.04	6.21	T	C	24	53.22	53.14	race 3
rs949365	Gm04	949365	6.25	0.05	7.49	A	C	12	61.56	53.14	race 3
rs46050545	Gm04	46050545	7.2	0.11	12.93	A	T	17	123.34	53.14	race 3
rs26781135	Gm05	26781135	7.21	0.04	9.78	C	T	53.58	145.55	58.23	race 4
rs33843704	Gm05	33843704	8.21	0.08	7.4	T	C	14	95.36	53.14	race 3
rs36404700	Gm07	36404700	10.7	0.13	10.03	G	A	13	92.45	53.14	race 3
rs8050006	Gm08	8050006	6.57	0.1	10.4	C	A	18	56.33	53.14	race 3
rs8050006	Gm08	8050006	9.11	0.16	14.29	C	A	7.53	56	53.14	race 4
rs41643371	Gm08	41643371	5.58	0.01	8.77	C	G	54.13	98.22	58.23	race 4
rs34608484	Gm09	34608484	7.55	0.05	9.26	C	A	20	78.26	53.14	race 3
rs33488383	Gm11	33488383	7.43	0.04	10.73	C	A	20	86.75	53.14	race 3
rs30581306	Gm14	30581306	7.76	0.05	12.62	C	A	50.54	115.44	58.23	race 4
rs43617172	Gm14	43617172	8.07	0.1	10.65	C	T	12	84.22	53.14	race 3
rs33704130	Gm16	33704130	8.41	0.07	11.66	A	C	24	114	53.14	race 3
rs33704130	Gm16	33704130	6.47	0.07	12.66	A	C	53	124.14	58.23	race 4
rs17238884	Gm17	17238884	7.19	0.42	13.53	T	A	53.65	87.47	58.23	race 4
rs1643660	Gm18	1643660	7.13	0.1	15.4	G	C	18	25	53.14	race 3
rs2085816	Gm20	2085816	6.1	0.48	6.85	G	A	23	87	53.14	race 3

1: race 3 equals to Hg type 0; race 4 equals to Hg type 1,2,3,5,7

### Beneficial allele evaluation of peak SNP and genetic architecture of significantly associated genomic regions

To confirm the beneficial allele of each peak SNP associated with SCN resistance, the average FI value of the soybean accessions that carried each allele of peak SNPs were calculated. The result indicated that the average FI values of accessions with resistant alleles were significantly lower than that of the accessions with susceptible alleles for all 19 association signals. They were also lower than the average FI value of the whole association panel (Table 1). Therefore, these resistant alleles could be useful for marker-assistant selection (MAS) of SCN resistance.

**Fig. 5:**
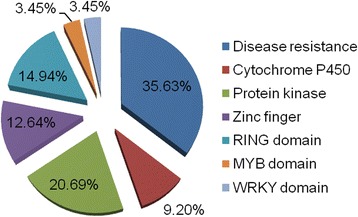
Main gene families in the flanking genomic regions of peak SNP

The candidate genes related to the identified loci against SCN Hg Type 0 and Hg Type 1.2.3.5.7 was further evaluated. Gene models that located in 200 Kbp genomic region upstream and downstream of each peak SNP in the reference soybean genome (Wm82.a2.v1, see ‘www. phetozome.net’) were considered to be resistance gene candidates since the mean LD decay distance of soybean genome was around 200 Kbp according to the present study and the reports of Hwang *et al.* (~360 Kbp) and Lam *et al.* (~150Kbp) [37, 45]. A total of 395 soybean gene models were found in the flanking regions of each peak SNP. Of these gene models, 64 genes had no functional annotation. Fourteen genes belonged to the domains of unknown function families. Among the other 317 gene models, several genes encoded serine/threonine protein kinases known to be involved in plant disease response. Genes encoding leucine-rich repeat-containing proteins, which might function in plant disease resistance pathways in response to a variety of external stimuli from pathogens, were also identified. Five other domain types were common including cytochrome P450s, protein kinases, zinc fingers, RING domains, MYB and WRKY families (Fig. 5). Of these gene families, there were five clusters of genes with disease resistance protein and/or LRR protein domains that were found in both side of rs33704130 on Gm16 that was associated with the resistance to both Hg Type 0 and Hg Type 1.2.3.5.7. The Hg Type 0 resistance loci (rs46050545 on Gm04, rs33843704 on Gm05, rs33488383 on Gm11 and rs43617172 on Gm14), as well as Hg Type 1.2.3.5.7 resistance locus (rs172388849 on Gm17) were tightly linked with the genes of RING/U-box superfamily protein or RING-H2 finger C1A gene. The distance of each RING gene to their corresponding peak SNP ranged from 8.9 Kbp to 161.7 Kbp with an average distance of 59.1 Kbp. It could be inferred that the RING/U-box superfamily protein might be involved in the disease resistance responsive to Hg Type 0 and Hg Type 1.2.3.5.7.

## Discussion

The precisely identifying and positioning of QTL are crucial for successful MAS. Up to now, many QTL associated with SCN resistance have been effectively tagged with different molecular marker systems, including SSR, RAPD, RFLP and SNP [12, 14, 19, 46–48]. However, no sequencing based mapping like GWAS for QTL or genes underlying the resistance to SCN has been reported so far. In the present study, the GWAS mapping approach and sequence based SNP maps with a large and diverse sample of soybean germplasm collection were used to identify the quantitative trait loci associated with the resistance to SCN Hg Type 0 (race 3) and Hg Type 1.2.3.5.7 (race 4). A total of 19 QTL were identified.

Among the 12 QTN (nucleotides) associated with QTL underlying resistance to SCN Hg Type 0, one QTN (rs8050006) was identified on chromosome 8 (Gm 08, LG A2), where a known and major QTL region associated with *Rhg4* locus had been commonly reported before [12, 17, 47, 49–51]. Concibido *et al.* [52] reported that a QTL near the *Rhg4* locus could explain 15 % of the total phenotypic variation in PI 209332 to SCN Hg Type 0. The *Rhg4* locus was confirmed as one of the major QTL controlling soybean resistance against SCN Hg Type 0 [50, 53]. Moreover, Kazi *et al.* and Ferreira *et al.* [17, 19, 49, 54] confirmed the effect of *Rhg4* on resistance to race 3 and 14, while *Rhg4* was less associated with resistance to races 1, 2, 4 and 5. However, this locus was also detected to be related to the resistance to Hg Type 1.2.3.5.7 (race 4) in our study, indicating that it could underlie SCN resistance more than one races simultaneously and might facilitate to breed new soybean varieties resistant to SCN race 3, 4 and 14.

In soybean, Gm 18 (LG G) was very important for SCN resistance. The *rhg1* loci, located on Gm 18, had been conformed to have an impact on the depression of SCN development in several resistance lines and provided SCN resistance in many commercial varieties from several resistance sources [12, 17, 19, 55–57]. For example, Concibido *et al.* [12] reported the recessive gene *rhg 1* that located on Gm 18 was identified to resistant to SCN race 3 and 14 as a major SCN resistance source. Similarly, Wu *et al.* [17] also detected a major QTL near *rhg1* with a relative large R^2^, resistant to SCN race 1, 2, 3, 5 and 14, respectively. Here, the SNP rs1643660, associated with the resistance to SCN Hg Type 0 (race 3), was detected to fall within the *rhg1* locus and was located in the 31 Kbp copy number variation (CNV) region of *Rhg1* locus. The SNP marker rs1643660 was 5.1 Kpb, 4.6 Kbp and 5.1 K bp distances from the three key genes (*Glyma.18 g022400, Glyma.18 g022500*) in CNV region [20]. However, the distance between SSR marker BARCSOYSSR_18_0090 and *rhg 1*-b was 67 Kbp in a fine mapping study of *rhg 1* locus that was reported by Kim *et al.* [58]. The distance from marker to causal genes in our study was much shorter than that reported by Kim *et al.,* indicating that better mapping result could be available from GWAS using SNP markers. The locus rs1643660 could be more effective for marker-assisted selection of lines resistant to SCN.

Additionally, most of the SNPs associated with the resistance to SCN Hg Type 0 (race 3) fell within the previously reported SCN resistant QTL intervals in the present study [17, 19, 47, 49, 52, 54, 59–63]. For example, two loci (rs949365 and rs46050545), resistant to SCN Hg Type 0 on Gm 04 (LG C1), were similar to the results reported by Yue *et al.* and Vuong *et al.* [61, 63]. In particular, the locus rs33704130 on Gm 16 (LG J) was detected to associate with the resistance to SCN Hg Type 0 (race 3) and Hg Type 1.2.3.5.7 simultaneously, which was similar to the results reported by Chang *et al.* [57].

A total of seven SNPs were associated with the resistance to SCN Hg Type 1.2.3.5.7 (race 4), and six of them were within the previously reported marker intervals [17, 52, 57, 61, 63, 64]. Of them, rs8050006 and rs33704130 were overlapped with the same loci associated with resistance to SCN Hg Type 0, suggesting that they could accelerate the development of soybean varieties with the resistance both to SCN Hg Type 0 and Hg Type 1.2.3.5.7.

In the present study, rs2085816 on Gm 20 (LG I), rs21804864 on Gm02 (LG D1b) and rs30581306 on Gm14 (LG B2) were the novel loci reported for SCN resistance. They might have the potential to be used in MAS for enhanced SCN Hg Type 0 and Hg Type 1.2.3.5.7 resistance.

Here, we mapped 19 QTL representing 17 genomic regions associated with soybean resistance to Hg Type 0 and/or Hg Type 1.2.3.5.7. More than 300 candidate genes were found in the linked region of these QTL, and as many as 25 % might have SCN response. Disease resistance-like proteins were found to be induced by different Hg Types of SCN in other studies too such as by Mazarei *et al.* and Matsye *et al.* [65, 66]. That suggested that disease resistance like proteins was a class of genes with broad-spectrum resistance to SCN. In addition, expression patterns of genes that encoded cytochrome P450, zinc finger protein, MYB domain protein, and WRKY DNA-binding protein that were mapped here were changed in SCN induced soybean roots [65–68]. SAUR-like genes has been suggested to encode short-lived nuclear proteins involved in auxin signaling by interacting with calmodulin [69, 70]. A report about auxin-responsive factor (ARF) in *Arabidopsis thaliana* described that double mutants of ARF gene showed a strong auxin phenotype that resulted in the absence of lateral root formation than single mutant expression. In addition, *Glyma.02g161600* that encoded the RING-H2 finger domain was the nearest gene to rs21804864 which is a novel locus associated with resistance to SCN. RING-H2 finger as an domain in E3 ubiquitin ligase was considered to participate in the degradation of proteins from the endoplasmic reticulum in Human [71]. E3 ubiquitin ligases was known to play an important role in mediating plant defence signaling [72]. Therefore, *Glyma.02g161600* with RING-H2 finger domain might be a new gene source of SCN resistance. However, many such genes alter the degree of susceptibility (60 < FI < 300) more than the degree of resistance (FI < 10).

## Conclusions

GWAS was successfully used to examine the genetic architecture of resistance to SCN Hg Types 0 and Type 1.2.3.5.7 in multiple genetic backgrounds. Nineteen associations were obtained for SCN resistance to the two Hg Types. The multiple beneficial alleles from resistant germplasm may be useful to breed varieties with improved resistance to SCN. Identification of causal gene(s) underlying SCN resistance may be assisted.

## Methods

### Evaluation of soybean resistance to HG types

Exactly 402 of the 440 accessions of soybean were collected from the soybean production areas between 19° N and 48° N of China. They included landraces and elite cultivars. The maturity groups of these germplasm were ranged from MG 000 to MG IX. Another 38 accessions were from the non-Chinese regions to represent the world-wide genetic diversity of *G. max*. Seeds of the soybean accessions were germinated in vermiculite and transplanted singly into 7.5 cm diameter clay pots when cotyledons opened. To evaluate the susceptibility to Hg Type 0 and Hg Type 1.2.3.5.7 the plants from each accession were transplanted into five pots. Plants were inoculated with 2,000 eggs and/or second-stage juveniles in a 10-ml suspension 2 days later. All plants were grown in a greenhouse at 25–28 °C. Thirty days after inoculation, the cysts and females were collected and counted. The female index was calculated as follows: FI = (number of cysts and females on detected plant)/(average number of cysts and females on ‘Lee 68’) × 100. FI > 10 was assigned “+” and FI < 10 was assigned “-” [73]. Each accession for each treatment contained five plants. A complete randomized design was used with three replicates. Each experiment was repeated twice.

### Genotyping of soybean germplasm collection

Genomic DNA was isolated from fresh leaves of a single plant per accession using the methods described by Wu *et al.* [74]. Subsequently, the DNA was analyzed by Specific Locus Amplified Fragment (SLAF) sequencing (SLAF-seq) [39]. Sequencing libraries of each accession were constructed through in *silico* digestion prediction and double digestion with enzymes *Mse*I and *Hae*III which could digest soybean genome DNA into more than 50,000 sequencing tags with 300–500 bp in length. These tags were evenly distributed in unique regions of genome. The 45 bp sequence read at both ends of each library was generated by Illumina Analyser II using a barcode approach to identify each sample.

The Short Oligonucleotide Alignment Program 2 (SOAP2) was used to map raw paired-ends reads onto the reference genome (Glycine_ max_Williams_82 8x Release v1.01) [75]. The SLAF groups were generated by reads that mapped to the same position. Because of sequencing errors, some reads appeared to be unique singletons. These reads were coalesced the sort of reads with lower quality but higher depth by allowing 1–3 mismatches. If an accession was only partially digested by the restriction enzymes, some reads mapped to reference genome should have overlaps with two SLAF tags. Such reads were assigned to both SLAF tags in that accession. A total of 60 thousand high quality SLAF tags were obtained from each of the 440 genotypes.

In SNP calling, minor allele frequencies (MAF) threshold was set at 0.04. If depth of minor allele was larger than 1/3 of sample total depth, the genotype were regarded as heterozygous.

### Population structure evaluation and LD analysis

Population structure of soybean natural population was conducted using principle component analysis (PCA) approach in GAPIT software package [76]. LD between pairs of SNPs was estimated by using squared allele frequency correlations (r^2^) in TASSEL version 3.0 [77]. Only SNPs with a MAF more than 0.04 and less than 10 % missing data were used to estimate LD. In contrast to the GWAS, missing SNP genotypes were not imputed with the major allele before LD analysis. Parameters in the program included MAF (≥0.04) and the integrity of each SNPs (≥80 %).

### Genome-wide association analysis

A total of 35,760 SNPs from soybean accessions were used for association analysis with Compressed Mixed Linear Model (MLM) in GAPIT [76]. The p values were adjusted with the Bonferroni method at α ≤ 0.01 and 0.05 level (corresponding *p* ≤ 2.70 × 10^−7^ and 1.35 × 10^−6^, respectively) and was used as the threshold to determine whether a significant association existed [78]. Candidate genes that located within 200 Kbp around the 5′ and 3′ direction of peak SNP were identified.

## Additional files

Additional file 1:
**Sources and distributions of 440 soybean accessions.** (PDF 433 kb) Additional file 2:
**SNP list of 440 soybean accessions.** (ZIP 2495 kb) Additional file 3:
**The overlap or linkage relationship of peak SNP and known QTL associated with SCN resistance.** (PDF 242 kb)
