# Supplementary material for: Genetic characteristics of soybean resistance to HG type 0 and HG type 1.2.3.5.7 of the cyst nematode analyzed by genome-wide association mapping
Source: BMC Genomics. 2015 Aug 13;16(1):598. doi: 10.1186/s12864-015-1800-1 (PMC4542112; doi:10.1186/s12864-015-1800-1)
Supplement: Additional file 1: — Sources and distributions of 440 soybean accessions. (PDF 433 kb) [file 12864_2015_1800_MOESM1_ESM.pdf]

Additional file 1 Sources and distributions of 440 soybean accessions.

No.	Name of accession	Country	Latitude (°N)	Longitude (°W)	Resistance to Hg type 0	Resistance to Hg type 1.2.3.5.7
1	AC033	China	22.715	113.325	MS	VS
2	Aika166	Rumania	44.23	26.1	MS	VS
3	Amsoy	America	41.83	92.905	MS	VS
4	B1361	China	48.29	128.08	MS	MS
5	B1484	China	48.29	128.08	MS	VS
6	B1873	China	48.29	128.08	MS	VS
7	B4834	China	48.29	128.08	MR	MR
8	Baiqidou	China	40.845	121.995	MS	VS
9	Baiqiu1	China	25.76	117.95	MS	VS
10	Bayfield	Canada	43.4	79.25	MR	VS
11	BD14	China	48.29	128.08	MS	VS
12	BD16	China	48.29	128.08	MS	VS
13	BF9	China	48.29	128.08	MS	MS
14	Big black	China	36.225	118.31	VS	VS
15	Big green seed	China	25.76	117.95	MS	MS
16	Big hair	China	31.125	112.14	VS	VS
17	Big yellow seed	China	25.76	117.95	MS	MR
18	Big Zhu	China	27.165	115.81	MS	MR
19	Bigwu	China	23.385	108.165	MS	MR
20	Binhaibaihua	China	32.825	118.875	MR	MS
21	Binhaihangkezi	China	32.825	118.875	MR	MS
22	Black	China	35.385	108.22	MR	MS
23	Black2	China	39.19	116.285	VR	VR
24	Bmoshi	China	43.35	126.285	MS	VR
25	Bragg	America	37.15	119.52	VS	VS
26	BX23	China	29.275	120	MS	MS
27	Cateye	China	37.34	100.295	MS	VS
28	Cdou1	China	37.34	84.79	MS	VS
29	Century l	America	37.15	119.52	VS	VS
30	Chamoshidou	China	43.35	126.285	MS	VS
31	Changsha2	China	27.42	111.365	MS	MS
32	Chanshani	China	27.42	111.365	MS	MS
33	Charleston	America	41.83	92.905	VS	MS
34	Chundou8	China	30.11	102.76	MS	MS
35	Chuxiu	China	32.825	118.875	MS	MS
36	Clark	America	34.65	91.915	VS	VS
37	Cmoshi	China	43.35	126.285	MR	VR
38	Conrad	America	41.83	92.905	VS	MS
39	Cqin	China	31.895	116.955	MS	MS
40	Cse	China	43.35	126.285	MS	MR
41	Cshiyuehang	China	30.11	102.76	MS	MS
42	Dahang1	China	37.385	112.235	MS	MS
43	Daidou1	China	31.125	112.14	MS	VS

44	Dami	China	31.125	112.14	MS	MS
45	Daqin	China	29.275	120	MS	MS
46	Datenhei	China	39.19	116.285	VR	VR
47	DN L-13	Canada	43.4	79.25	MS	VS
48	DN07-909	China	48.29	128.08	VS	MS
49	DN1068	China	48.29	128.08	VS	MR
50	DN163	China	48.29	128.08	MS	MS
51	DN42	China	48.29	128.08	VS	VS
52	DN43	China	48.29	128.08	MR	VR
53	DN44	China	48.29	128.08	MS	VS
54	DN47	China	48.29	128.08	MS	MS
55	DN48	China	48.29	128.08	MS	VS
56	DN49	China	48.29	128.08	MS	MS
57	DN56	China	48.29	128.08	MS	MS
58	DN594	China	48.29	128.08	MS	MS
59	DN93046	China	48.29	128.08	MS	MS
60	Domaka Tolisa	Yugoslavia	46.3	14.3	MS	VS
61	Donshan	China	31.125	112.14	MS	VS
62	Douludou	China	43.35	126.285	MS	VS
63	Dsmall pod	China	32.825	118.875	MS	MS
64	Dunajika	Russia	61.5	98	MS	MS
65	Duomala	Yugoslavia	46.3	14.3	MS	VS
66	DY2004-5	China	43.35	126.285	MS	VS
67	Dyer	America	35.8	78.8	MR	VS
68	Early18	China	40.145	116.275	MS	VS
69	Edou7	China	31.125	112.14	MS	VS
70	Electron	Canada	43.4	79.25	MS	MR
71	Erjizao-2	China	26.75	106.355	MS	VS
72	F310	China	25.76	117.95	MS	VS
73	Fall bean	China	25.76	117.95	MS	MR
74	Fall2	China	25.76	117.95	MS	VS
75	Fenchenzao	China	27.165	115.81	MS	MR
76	Fendiyellow	China	43.35	126.285	MS	VS
77	Fendou72	China	37.385	112.235	MS	MR
78	Fenshou6	China	48.29	128.08	MS	MS
79	Forrest	America	35.8	78.8	VS	MR
80	Fu8	China	23.385	108.165	MS	VS
81	Fudou234	China	25.76	117.95	MS	MS
82	Fwo	China	29.275	120	MS	VS
83	FZmoshidou	China	48.29	128.08	MR	MR
84	G early1	China	23.385	108.165	MS	MS
85	G0118-1	China	23.385	108.165	MS	VS
86	G5	China	27.165	115.81	MS	MS
87	Gandou4	China	27.165	115.81	MS	VS
88	Ganyuhai	China	27.165	115.81	VS	VS
89	GC1	China	23.385	108.165	MS	VS
90	Gon13	China	30.11	102.76	MS	MS
91	Gonye04-141	China	43.35	126.285	MS	MS

92	Green coat	China	27.165	115.81	MS	VS
93	Green6	China	36.225	118.31	MS	VS
94	Greengun	China	35.385	108.22	MS	MS
95	Greyheidou	China	37.385	112.235	VR	VR
96	GX2	China	23.385	108.165	VS	MS
97	Gxijangdou	China	30.11	102.76	VS	MR
98	H04-1824	China	48.29	128.08	VS	MR
99	H05-31	China	48.29	128.08	VS	VS
100	H05-991	China	48.29	128.08	VS	MR
101	Habanqin	China	29.275	120	VS	MR
102	Haidedou	China	32.825	118.875	VS	MR
103	Hangbaodou	China	40.845	121.995	VS	VS
104	Hangdou2	China	37.385	112.235	VS	MR
105	Hanggan	China	37.385	112.235	VS	MR
106	Hangmao	China	27.42	111.365	VS	MR
107	Hanyanheidou	China	30.11	102.76	VS	MR
108	Harosoy	America	35.8	78.8	VS	MR
109	Hartwig	America	35.8	78.8	VR	VR
110	Harvest10	China	48.29	128.08	VS	VS
111	HC1	China	22.715	113.325	VS	MR
112	HC6	China	22.715	113.325	VS	MR
113	HD3	China	31.895	116.955	VS	MR
114	Hedou	China	22.715	113.325	VS	MR
115	Hedou12	China	36.225	118.31	VS	MR
116	Heersong2	Ukraine	50.28	30.29	VS	MS
117	Heidou10252	China	35.385	108.22	VR	MR
118	Heidou10270	China	35.385	108.22	VR	MS
119	Heidou3	China	22.715	113.325	VS	MR
120	Heihehangdou	China	48.29	128.08	VS	MR
121	Heilongjiang41	China	48.29	128.08	VS	MR
122	Heimodou	China	43.35	126.285	VR	VR
123	Heinong33	China	48.29	128.08	VS	MR
124	heipidou	China	31.895	116.955	VS	MS
125	Heiwudou	China	19.1	109.71	VS	VS
126	HF11	China	48.29	128.08	VS	VS
127	HF25	China	48.29	128.08	VS	VS
128	HF29	China	48.29	128.08	VS	MS
129	HF35	China	48.29	128.08	VS	MS
130	HF37	China	48.29	128.08	VS	VS
131	HF45	China	48.29	128.08	VS	MR
132	HF47	China	48.29	128.08	VS	MR
133	HF50	China	48.29	128.08	VS	VS
134	HF52	China	48.29	128.08	VS	MS
135	HF55	China	48.29	128.08	VS	MS
136	HH05-1676	China	48.29	128.08	VS	VS
137	HH18	China	48.29	128.08	VS	VS
138	HH38	China	48.29	128.08	VS	VS
139	HH45	China	48.29	128.08	VS	MS

140	HH48	China	48.29	128.08	VS	MS
141	Hhang	China	35.385	108.22	VS	MS
142	Hill	America	35.8	78.8	VS	MS
143	HJ03-286	China	44.49	111.695	VS	MS
144	HJ04-528	China	44.49	111.695	VS	VS
145	HJ2	China	48.29	128.08	VS	MS
146	HJ423	China	44.49	111.695	VS	VS
147	HJ4403	China	48.29	128.08	VS	MS
148	HN1	China	27.42	111.365	VS	MS
149	HN37	China	48.29	128.08	VS	MR
150	HN44	China	48.29	128.08	VS	MS
151	HN48	China	48.29	128.08	VS	VS
152	HN51	China	48.29	128.08	VS	VS
153	HN55	China	48.29	128.08	VS	MS
154	Honhedou	China	31.125	112.14	VS	MS
155	Huadou20	China	33.725	113.3	VS	VS
156	Huaiyin	China	32.825	118.875	VS	MS
157	Huajian1	China	48.29	128.08	VS	VS
158	Huasedou	China	31.125	112.14	VS	MS
159	Hubuzhi	China	37.385	112.235	VR	VS
160	Huzimao	China	31.125	112.14	VS	MS
161	HX1	China	22.715	113.325	VS	MS
162	HX3	China	22.715	113.325	VS	MS
163	HX8	China	22.715	113.325	VS	MS
164	HYoutai	China	43.35	126.285	VS	VS
165	J06B7	China	39.19	116.285	MR	VS
166	J100	China	43.35	126.285	VS	MS
167	J17	China	39.19	116.285	VS	VS
168	J89	China	43.35	126.285	VS	VS
169	J94	China	43.35	126.285	MR	MS
170	Janghang	China	35.385	108.22	VS	MR
171	JD12	China	39.19	116.285	MR	MR
172	JD18	China	39.19	116.285	VS	MR
173	JD33	China	40.845	121.995	MR	MR
174	Jheidou	China	37.385	112.235	VR	VR
175	Jidou9	China	39.19	116.285	VS	MR
176	Jin1265	China	37.385	112.235	MR	MR
177	Jin13	China	39.19	116.285	VS	MS
178	Jin38	China	37.385	112.235	MR	MR
179	Jin78	China	37.385	112.235	VS	MS
180	Jindou21	China	37.385	112.235	MR	MS
181	Jinhang35	China	31.125	112.14	VS	MR
182	Jishanpu	China	48.29	128.08	MR	MR
183	Jiyan1	China	43.35	126.285	VS	MR
184	JL30	China	43.35	126.285	MR	MR
185	JL47	China	43.35	126.285	VS	VS
186	JLcaha	China	43.35	126.285	MR	MS
187	JN20	China	43.35	126.285	VS	VR

188	JN21	China	43.35	126.285	MR	MR
189	Jumbo emas	Japan	35.41	139.44	VS	MR
190	JY46	China	37.385	112.235	MR	MR
191	JZ4	China	40.845	121.995	VS	VS
192	K04-8579	China	48.29	128.08	MR	VS
193	K22	China	48.29	128.08	VS	MR
194	K29	China	48.29	128.08	MR	MR
195	K30	China	48.29	128.08	VS	MS
196	K4seed	China	48.29	128.08	MR	MR
197	KB1	China	48.29	128.08	VS	VR
198	Keburi	Japan	35.41	139.44	MR	MR
199	Ken14	China	48.29	128.08	VS	VS
200	Ken15	China	48.29	128.08	MR	MR
201	Ken18	China	48.29	128.08	VS	MS
202	Ken23	China	48.29	128.08	MR	MS
203	KX3	China	40.145	116.275	VS	VS
204	L-10	Canada	43.4	79.25	VR	VR
205	L-79	Canada	43.4	79.25	MR	MS
206	L98072	China	40.845	121.995	VS	MR
207	Laoxan1	China	40.845	121.995	MS	MR
208	LD11	China	36.225	118.31	VS	MR
209	LD23	China	40.845	121.995	MS	VS
210	LD3	China	40.845	121.995	VS	MS
211	Lee	America	35.8	78.8	MS	VS
212	Lhang	China	22.715	113.325	VS	MR
213	Liu3	China	23.385	108.165	MS	MR
214	Liuyuexian	China	32.825	118.875	VS	MR
215	LN2	China	40.845	121.995	MS	MS
216	Long7	China	23.385	108.165	VS	MS
217	Lonqan	China	48.29	128.08	MS	MR
218	LP03-11	China	48.29	128.08	VS	VR
219	Lu4	China	36.225	118.31	MS	MS
220	Lupihuang	China	37.385	112.235	VS	MS
221	LX1	China	48.29	128.08	MS	MR
222	M19	China	44.49	111.695	VS	MS
223	M9449	China	31.895	116.955	MS	VS
224	M9793-1	China	31.895	116.955	VS	MS
225	Maple Arrow	Canada	43.4	79.25	MS	VS
226	MD14	China	44.49	111.695	VS	MR
227	MD21	China	44.49	111.695	VS	VS
228	MD9	China	44.49	111.695	VS	VS
229	Meidou	China	31.125	112.14	VS	MS
230	MF1	China	48.29	128.08	VS	VS
231	Mousepi	China	35.385	108.22	VS	MS
232	Mqin2	China	22.715	113.325	VS	MR
233	Mutant30	China	40.145	116.275	VS	MR
234	Mutant446	China	39.19	116.285	VS	MR
235	N989445A	America	43.4	79.25	VS	MR

236	Nanganqin	China	39.19	116.285	VS	MS
237	NF11	China	48.29	128.08	VS	MS
238	NF15	China	48.29	128.08	VR	MS
239	Nigeria5	Nigeria	9.12	7.11	VS	MS
240	Nmeidou	China	37.185	105.78	VS	MR
241	NN30	China	32.825	118.875	VS	MR
242	Nova	Italy	41.54	12.3	VS	MR
243	Panstone	China	29.275	120	VS	MS
244	PD451	China	25.76	117.95	VS	MS
245	Peixianyoudou	China	32.825	118.875	VS	MR
246	Peking	America	40.42	74	VR	MR
247	Phangkezi3	China	30.11	102.76	VS	MS
248	Phonmao	China	32.825	118.875	VS	MS
249	PI437654	America	41.83	92.905	VR	VR
250	PI486355	America	41.83	92.905	VS	MS
251	Pindin	China	36.225	118.31	VS	MS
252	Ping black	China	36.225	118.31	VS	MS
253	Pixanpurple	China	32.825	118.875	VR	VR
254	Pixianxiaodou	China	30.11	102.76	VS	MS
255	Pohang	China	22.715	113.325	VS	MS
256	Purple4	China	39.19	116.285	VS	MS
257	Qan253	China	25.76	117.95	VS	MS
258	Qan7	China	25.76	117.95	VS	MS
259	Qan8	China	25.76	117.95	VS	MS
260	Qanban11	China	25.76	117.95	VS	MS
261	Qbayuehang	China	30.11	102.76	VS	MS
262	QD31	China	36.225	118.31	VS	MS
263	QH25	China	36.225	118.31	VS	MS
264	QH28	China	36.225	118.31	VS	MS
265	QH30	China	36.225	118.31	VS	MS
266	QH32	China	36.225	118.31	VS	MS
267	QH33	China	36.225	118.31	VS	MS
268	Qhei	China	48.29	128.08	VS	MS
269	Qiandou5	China	39.19	116.285	VS	MS
270	Qindou	China	39.19	116.285	VS	MS
271	Qinhei	China	48.29	128.08	VS	VS
272	Qisiwa	China	36.225	118.31	VS	VS
273	Qshuidou	China	30.11	102.76	VS	MS
274	Red Zhu	China	27.42	111.365	VS	MS
275	Renfenhang	China	22.715	113.325	VS	MS
276	Rijinqin	China	27.165	115.81	VS	VS
277	S02-339	China	48.29	128.08	MS	MS
278	S03-3046	China	48.29	128.08	MS	VS
279	S03-3952	China	48.29	128.08	MS	MS
280	S04-5804	China	48.29	128.08	MS	VR
281	S04-6018	China	48.29	128.08	MS	VS
282	S05-7304	China	48.29	128.08	MS	MR
283	S28	China	48.29	128.08	MS	VS

284	S29	China	48.29	128.08	MS	VS
285	S30	China	48.29	128.08	MS	VS
286	Saugust	China	27.165	115.81	MS	VS
287	Semi-dwarf	China	48.29	128.08	MS	VS
288	Sfentai	China	30.11	102.76	MS	VS
289	Sgreen	China	25.76	117.95	MS	VS
290	SH10	China	48.29	128.08	MS	MS
291	Shan95	China	33.725	113.3	MS	VS
292	shandong69	China	37.385	112.235	MS	VS
293	Shannin7	China	36.225	118.31	MS	VS
294	Shanzibai	China	31.125	112.14	MS	VS
295	Shaxin	China	27.165	115.81	MS	VS
296	Shichenghongdou	China	33.725	113.3	MS	VS
297	Shishengchangye	Japan	35.41	139.44	MS	VS
298	Short early	China	32.825	118.875	MS	VS
299	Short Nidou	China	27.42	111.365	MS	VS
300	Sidou2	China	32.825	118.875	MS	VS
301	Silicao	China	32.825	118.875	MS	VS
302	Single yellow	China	29.275	120	MS	VS
303	Slosidou	China	30.11	102.76	MS	VS
304	Small Moshi	China	48.29	128.08	MS	VR
305	Small seed9	China	48.29	128.08	MS	-
306	Small yellow	China	37.385	112.235	MS	VS
307	SN1	China	48.29	128.08	MS	VS
308	SN14	China	48.29	128.08	MS	VS
309	SN25	China	48.29	128.08	MS	VS
310	Spindinhang	China	32.825	118.875	MS	MR
311	Star4	China	39.19	116.285	MS	VS
312	Stone	China	39.19	116.285	MS	MR
313	Sudou	China	29.275	120	MS	MS
314	Sui20	China	48.29	128.08	MS	MR
315	Suinong4	China	48.29	128.08	MS	VS
316	Suinong8	China	48.29	128.08	MS	MS
317	Summerhei	China	37.385	112.235	MS	MS
318	Swan	China	36.225	118.31	MS	MR
319	Swandou	China	37.385	112.235	MS	MS
320	Swu	China	25.76	117.95	MS	MS
321	Tangen	China	29.275	120	MS	MS
322	TD50	China	40.845	121.995	MS	MS
323	TD51	China	40.845	121.995	MS	MS
324	TD52	China	40.845	121.995	MS	VS
325	TD54	China	40.845	121.995	MS	VS
326	TD58	China	40.845	121.995	MS	MS
327	Tejia	China	40.845	121.995	VS	VS
328	Tenzai	China	25.76	117.95	VR	MS
329	TF8	China	40.845	121.995	MS	VS
330	TGreen	China	32.825	118.875	MS	MS

331	Theidou	China	32.825	118.875	MS	MS
332	Thon	China	25.76	117.95	VS	MS
333	Tiefeng31	China	40.845	121.995	MS	VS
334	Tiger	China	29.275	120	MS	MS
335	TJ4	China	43.35	126.285	MS	MS
336	TN13	China	43.35	126.285	MS	MS
337	Tshort	China	32.825	118.875	MS	MS
338	V111-4	China	43.35	126.285	MS	MS
339	Vectory3	China	40.845	121.995	MS	MR
340	W16	China	31.895	116.955	MR	MR
341	W24	China	31.895	116.955	MS	MR
342	Wenfan7	China	40.845	121.995	MS	MS
343	White hair	China	25.76	117.95	MS	MS
344	Williams82	America	41.83	92.905	VS	VS
345	DN L-100	China	48.29	128.08	MS	MS
346	Winter	China	30.11	102.76	MS	MS
347	Wudou	China	27.165	115.81	MS	MS
348	bigwudou	China	31.125	112.14	VS	MS
349	X12	China	32.825	118.875	MS	MS
350	Xamoshidou	China	39.19	116.285	MS	VS
351	XC10	China	27.42	111.365	MS	VS
352	XC23	China	27.42	111.365	MS	VS
353	XC24	China	27.42	111.365	MS	VS
354	XCdou13	China	27.42	111.365	MS	VS
355	XCdou18	China	27.42	111.365	MS	VS
356	XCdou19	China	27.42	111.365	MS	VS
357	Херсohскaя2	Former Soviet Union	61.5	98	MS	VS
358	Xian2	China	48.29	128.08	VR	MS
359	Xian3	China	48.29	128.08	MS	MS
360	Xiaomidou	China	36.225	118.31	MS	VS
361	Xinyuqing	China	27.165	115.81	MS	MS
362	Xudou3	China	33.725	113.3	MS	VS
363	Xuqandou	China	32.825	118.875	MS	VS
364	Y15	China	33.725	113.3	MS	VS
365	Y16	China	33.725	113.3	MS	VS
366	Y27	China	33.725	113.3	MS	VS
367	Y28	China	33.725	113.3	MS	VS
368	Yanbojin	China	48.29	128.08	MS	MS
369	Yandou	China	39.19	116.285	MS	VS
370	Yapiche	China	48.29	128.08	MS	MR
371	YBig seed	China	32.825	118.875	MS	VS
372	YD17	China	33.725	113.3	MS	VS
373	YD23	China	33.725	113.3	MS	VS
374	Yhang	China	37.34	84.79	MS	VS
375	Yheidou	China	37.385	112.235	VR	VR
376	YJ5	China	36.225	118.31	MR	VS
377	You01-65	China	31.125	112.14	MR	VS

378	Youchun05-4	China	31.125	112.14	MR	VS
379	Youchun05-8	China	31.125	112.14	MR	VS
380	Z00-683	China	40.145	116.275	VS	VS
381	Z03-5179	China	40.145	116.275	MR	MS
382	Z03-5334	China	40.145	116.275	VS	VS
383	Z03-5355	China	40.145	116.275	MR	VS
384	Z03-5363	China	40.145	116.275	VS	VS
385	Z05-15	China	40.145	116.275	MR	VS
386	Z10	China	40.145	116.275	VS	MR
387	Z13	China	40.145	116.275	MR	VR
388	Z27	China	40.145	116.275	VS	VR
389	Z30	China	40.145	116.275	MR	VS
390	Z32	China	40.145	116.275	VS	VS
391	Z33	China	40.145	116.275	MR	VS
392	Z35	China	40.145	116.275	VS	MS
393	Z37	China	40.145	116.275	MR	VS
394	Z6	China	40.145	116.275	MR	VS
395	Z7016	China	33.725	113.3	VS	VS
396	Z8	China	40.145	116.275	MR	VS
397	Z8516	China	33.725	113.3	VS	VS
398	Z92116	China	33.725	113.3	MR	VS
399	Z9525	China	33.725	113.3	VS	VS
400	Z97196	China	33.725	113.3	MR	VS
401	Z9805	China	33.725	113.3	VS	VS
402	ZC2	China	29.275	120	MR	VS
403	ZC3	China	29.275	120	VS	VS
404	ZD02-281	China	33.725	113.3	MR	VS
405	ZD107	China	33.725	113.3	VS	VS
406	ZD11	China	33.725	113.3	MR	VS
407	ZD17	China	33.725	113.3	VS	VS
408	ZD7501	China	33.725	113.3	MR	MR
409	Zechan yellow	China	33.725	113.3	VS	VS
410	Zhidou	China	23.385	108.165	MR	-
411	Zhong03-5373	China	40.145	116.275	VR	VR
412	Zhong03-5413	China	40.145	116.275	VS	-
413	Zhong20	China	40.145	116.275	-	VS
414	Zhong24	China	40.145	116.275	-	-
415	Zhong95-5388	China	40.145	116.275	-	-
416	Zhonghuang35	China	40.145	116.275	-	MR
417	Zhonte1	China	40.145	116.275	-	-
418	ZJ4032	China	40.145	116.275	-	-
419	ZJ4033	China	40.145	116.275	-	-
420	Zliuyue	China	30.11	102.76	-	-
421	Zuoxuan1	China	36.225	118.31	-	-
422	297	Canada	43.4	79.25	MR	MS
423	760	Canada	43.4	79.25	MR	MR
424	18629	China	48.29	128.08	VS	MR
425	2340322	China	30.11	102.76	MR	MR

426	10C320	China	40.145	116.275	VS	MR
427	10C373	China	40.145	116.275	MS	-
428	10NC831	China	40.145	116.275	MR	-
429	4seed	China	36.225	118.31	MS	-
430	4yellow	China	48.29	128.08	MS	VS
431	5 months	China	27.165	115.81	MS	-
432	60hanchang	China	40.845	121.995	VS	MS
433	7803green	China	23.385	108.165	MS	-
434	84-70	China	31.125	112.14	MS	-
435	85-140	China	40.845	121.995	MS	VS
436	8yellow	China	29.275	120	MS	VS
437	9mousehang	China	29.275	120	MS	VS
438	Dongnong8004	China	48.29	128.08	MS	MS
439	Boige du	Germany	52.31	13.2	MS	MS
440	Z4066	China	33.73	113.3	VS	MS

Note: VR, MR, MS and VS represent very resistant, moderate resistant, moderate susceptible and very susceptible respectively according to the cutoff criterion of female index to each Hg type published by Schmitt and G. Shannon in 1992[1].

Reference

1. Schmitt, D. P., and G. Shannon: **Differentiating soybean cyst nematode race and resistance response of soybean**. *Crop Science* 1992, **32**:275–277.
