# Supplementary material for: Genetic characteristics of soybean resistance to HG type 0 and HG type 1.2.3.5.7 of the cyst nematode analyzed by genome-wide association mapping
Source: BMC Genomics. 2015 Aug 13;16(1):598. doi: 10.1186/s12864-015-1800-1 (PMC4542112; doi:10.1186/s12864-015-1800-1)
Supplement: Additional file 3: — The overlap or linkage relationship of peak SNP and known QTL associated with SCN resistance. (PDF 242 kb) [file 12864_2015_1800_MOESM3_ESM.pdf]

The overlap or linkage relationship of peak SNP and known QTL associated with SCN resistance.

| Peak SNP | Chromosome | Position | Hg type | QTL | Relation with peak SNP | Left flanking marker of QTL | Marker position | Right flanking marker of QTL | Marker position | Reference |
|---|---|---|---|---|---|---|---|---|---|---|
| rs8009636 | Gm01 | 8009636 | race 4 | SCN 26-2 | Linkage | Satt032 | 7326736 | Satt368 | 6916612 | Yue et al. 2001[1] |
| | | | | SCN 19-3 | Linkage | Sat_353 | 7701041 | Sat_413 | 354608 | Yue et al. 2001[2] |
| | | | | SCN 20-3 | Linkage | Sat_353 | 7701041 | Sat_413 | 354608 | Yue et al. 2001[2] |
| | | | | SCN 21-2 | Linkage | Sat_353 | 7701041 | Sat_413 | 354608 | Yue et al. 2001[2] |
| rs949365 | Gm04 | 949365 | race 3 | SCN 21-1 | Linkage | BARC-029425-06191 | 2206978 | BARC-030255-06839 | 1113971 | Yue et al. 2001[2] |
| | | | | SCN 36-6 | Linkage | Sat_140 | 5221426 | Sat_367 | 3243750 | Vuong et al. 2011[3] |
| rs46050545 | Gm04 | 46050545 | race 3 | SCN 36-7 | Linkage | Sat_207 | 42773904 | Satt294 | 40154806 | Vuong et al. 2011[3] |
| rs26781135 | Gm05 | 26781135 | race 4 | SCN 18-1 | Overlap | SOYNOD26A | 35100953 | Sat_410 | 18413059 | Yue et al. 2001[2] |
| rs33843704 | Gm05 | 33843704 | race 3 | SCN 18-1 | Overlap | SOYNOD26A | 35100953 | Sat_410 | 18413059 | Yue et al. 2001[2] |
| rs36404700 | Gm07 | 36404700 | race 3 | SCN 40-4 | Linkage | Satt250 | 39127792 | Satt551 | 37174014 | Ferreira et al. 2011[4] |
| rs8050006 | Gm08 | 8050006 | race 3 | SCN 39-8 | Overlap | BARC-013587-01169 | 10650702 | Sat_162 | 8279349 | Wu et al. 2009[5] |
| | | | | SCN 29-5 | Overlap | Satt424 | 10721660 | Sat_400 | 7885686 | Guo et al. 2006[6] |
| | | | | SCN 9-3 | Overlap | Satt424 | 10721660 | Sat_212 | 9166013 | Mahalingam 1995[7] |
| | | | | SCN 42-1 | Overlap | AW132402 | 11868919 | Sat_400 | 7885686 | Ariagada et al. 2012[8] |
| | | | | SCN 1-1 | Overlap | Satt589 | 5175904 | Satt480 | 4211667 | Concibido et al. 1994[9] |
| | | | | cqSCN-002 | Overlap | Sat_400 | 7885686 | BARC-011635-00314 | 6804182 | Mattews et al. 1998[10] Prabhu et al. 1999[11] |

| | | | | | | | | | | |
|---|---|---|---|---|---|---|---|---|---|---|
| | | | | | | | | | | Meksem et al. 2001B[12] |
| | | | | | | | | | | Meksem et al. 2001C[13] |
| | | | | | | | | | | Yuan et al. 2002[14] |
| | | | | | | | | | | Meksem et al. 2001A[15] |
| | | | | SCN 33-2 | Overlap | Satt632 | 8219188 | Sat_406 | 3987571 | Guo et al. 2006[6] |
| | | | | SCN 36-1 | Overlap | Satt632 | 8219188 | Satt315 | 6751511 | Vuong et al. 2011[3] |
| | | | | SCN 36-2 | Overlap | Satt632 | 8219188 | Satt315 | 6751511 | Vuong et al. 2011[3] |
| | | | | SCN 40-1 | Overlap | Sat_157 | 8349427 | BARC-011635-00314 | 6804182 | Ferreira et al. 2011[4] |
| | | | | SCN 27-2 | Overlap | BARC-038291-07245 | 8379940 | BARC-011635-00314 | 6804182 | Meksem et al. 2001C[13] |
| | | | | SCN 3-1 | Overlap | BARC-038291-07245 | 8379940 | BARC-011635-00314 | 6804182 | Webb et al. 1995[16] |
| | | | | SCN 9-2 | Overlap | BARC-038291-07245 | 8379940 | BARC-011635-00314 | 6804182 | Mahalingam 1995[7] |
| | | | | SCN 13-2 | Overlap | Sat_212 | 9166013 | GMENOD2B | 10190700 | Prabhu et al. 1999[11] |
| | | | | SCN 8-5 | Overlap | Sat_212 | 9166013 | GMENOD2B | 10190700 | Chang et al. 1997[17] |
| | | | | SCN 39-1 | Overlap | Sat_212 | 9166013 | Sat_162 | 8279349 | Wu et al. 2009[5] |
| | | | | SCN 41-4 | Overlap | Satt187 | 9199676 | GMENOD2B | 10190700 | Kazi et al. 2010[18] |
| | | | | SCN 41-5 | Overlap | Satt187 | 9199676 | GMENOD2B | 10190700 | Kazi et al. 2010 [18] |
| | | | | SCN 19-1 | Overlap | Sat_215 | 9211714 | Satt315 | 6751511 | Yue et al. 2001[1] |
| | | | | SCN 30-3 | Overlap | BARC-043119-08535 | 9997461 | BARC-013587-01169 | 10650702 | Brucker et al. 2005[19] |
| rs41643371 | Gm08 | 41643371 | race 4 | SCN 37-4 | Linkage | Sat_040 | 39048596 | Satt233 | 17297986 | Vuong et al. 2010[20] |
| rs34608484 | Gm09 | 34608484 | race 3 | SCN 39-5 | Overlap | Satt240 | 32913643 | Satt247 | 9265060 | Wu et al. 2009[5] |
| | | | | SCN 2-2 | Linkage | AQ851479 | 37892646 | Satt453 | 38360539 | Verling et al. 1996[21] |
| rs33488383 | Gm11 | 33488383 | race 3 | SCN 32-3 | Linkage | BE801538 | 38205774 | Satt453 | 38360539 | Ferdous et al. 2006[22] |
| | | | | SCN 29-10 | Linkage | Sat_331 | 38376841 | Satt359 | 36868221 | Guo et al. 2006[23] |

| | | | | | | | | | | |
|---|---|---|---|---|---|---|---|---|---|---|
| | | | | SCN 33-4 | Linkage | Sat_331 | 38376841 | BARC-021459-04106 | 38902483 | Guo et al. 2006[6] |
| | | | | SCN 33-6 | Linkage | Sat_331 | 38376841 | BARC-021459-04106 | 38902483 | Guo et al. 2006[6] |
| | | | | SCN 39-9 | Linkage | BARC-021459-04106 | 38902483 | Satt359 | 36868221 | Wu et al. 2009[5] |
| rs30581306 | Gm14 | 30581306 | race 3 | SCN 10-1 | Linkage | Sat_424 | 46983608 | Satt063 | 46705813 | Qiu et al. 1999[24] |
| rs33704130 | Gm16 | 33704130 | race 3 race 4 | SCN 38-3 | Overlap | Satt244 | 33327176 | Sat_350 | 30435381 | Chang et al. 2011[25] |
| | | | | SCN 28-2 | Linkage | Satt547 | 33538088 | Satt244 | 33327176 | Glover et al. 2004[26] |
| | | | | SCN 1-2 | Linkage | Sat_224 | 35386076 | BARC-030433-06867 | 33761218 | Concibido et al. 1994[9] |
| | | | | SCN 5-2 | Linkage | Sat_224 | 35386076 | BARC-030433-06867 | 33761218 | Concibido et al. 1997[27] |
| | | | | SCN 28-4 | Linkage | Satt431 | 35718413 | Satt244 | 33327176 | Glover et al. 2004[26] |
| | | | | cqSCN-003 | Linkage | Satt431 | 35718413 | Satt547 | 33538088 | Glover et al. 2004[26] |
| | | | | SCN 29-2 | Linkage | BARC-041173-07927 | 36278618 | Satt547 | 33538088 | Guo et al. 2006[23] |
| | | | | SCN 29-6 | Linkage | BARC-041173-07927 | 36278618 | Satt547 | 33538088 | Guo et al. 2006[23] |
| rs17238884 | Gm17 | 17238884 | race 4 | SCN 38-6 | Linkage | Satt082 | 19746628 | Satt514 | 18770794 | Chang et al. 2011[25] |
| rs1643660 | Gm18 | 1643660 | race 3 | SCN 40-2 | Overlap | Sat_210 | 1621167 | BARC-035305-07162 | 1726852 | Ferreira et al. 2011[4] |
| | | | | SCN 13-1 | Overlap | Sat_210 | 1621167 | BARC-035305-07162 | 1726852 | Prabhu et al. 1999[11] |
| | | | | SCN 4-1 | Overlap | Sat_210 | 1621167 | BARC-035305-07162 | 1726852 | Concibido et al. 1997[27] |
| | | | | SCN 5-1 | Overlap | Sat_210 | 1621167 | BARC-035305-07162 | 1726852 | Concibido et al. 1997[27] |
| | | | | SCN 6-1 | Overlap | Sat_210 | 1621167 | BARC-035305-07162 | 1726852 | Concibido et al. 1997[27] |
| | | | | SCN 8-3 | Overlap | Sat_210 | 1621167 | BARC-035305-07162 | 1726852 | Chang et al. 1997[17] |
| | | | | SCN 36-3 | Linkage | Satt309 | 1736253 | BARC-035305-07162 | 1726852 | Vuong et al. 2011[3] |

| | | | | | | |
|---|---|---|---|---|---|---|
| SCN 36-4 | Linkage | Satt309 | 1736253 | BARC-035305-07162 | 1726852 | Vuong et al. 2011[3] |
| SCN 36-5 | Linkage | Satt309 | 1736253 | BARC-035305-07162 | 1726852 | Vuong et al. 2011[3] |
| SCN 38-2 | Linkage | Satt309 | 1736253 | BARC-035305-07162 | 1726852 | Chang et al. 2011[25] |
| SCN 41-1 | Linkage | Satt309 | 1736253 | BARC-035305-07162 | 1726852 | Kazi et al. 2010[18] |
| SCN 33-3 | Overlap | Satt309 | 1736253 | Satt163 | 998312 | Guo et al. 2006[6] |
| SCN 33-5 | Overlap | Satt309 | 1736253 | Satt163 | 998312 | Guo et al. 2006[6] |
| SCN 41-2 | Overlap | Satt309 | 1736253 | Satt163 | 998312 | Kazi et al. 2010[18] |
| cqSCN-001 | Overlap | BARC-027452-06569 | 1788836 | Sat_210 | 1621167 | Mudge et al. 1997[28]<br>Yuan et al. 2002[14]<br>Meksem et al. 2001A[15] |
| SCN 39-3 | Linkage | BARC-027452-06569 | 1788836 | BARC-035305-07162 | 1726852 | Wu et al. 2009[5] |
| SCN 41-3 | Linkage | BARC-027452-06569 | 1788836 | BARC-035305-07162 | 1726852 | Kazi et al. 2010[18] |
| SCN 14-2 | Linkage | BARC-027452-06569 | 1788836 | BARC-035305-07162 | 1726852 | Meksem et al. 1999[29] |
| SCN 30-1 | Linkage | BARC-027452-06569 | 1788836 | BARC-035305-07162 | 1726852 | Brucker et al. 2005[19] |
| SCN 30-2 | Linkage | BARC-027452-06569 | 1788836 | BARC-035305-07162 | 1726852 | Brucker et al. 2005[19] |
| SCN 23-3 | Linkage | BARC-027452-06569 | 1788836 | Satt309 | 1736253 | Yue et al. 2001[1] |
| SCN 24-2 | Linkage | BARC-027452-06569 | 1788836 | Satt309 | 1736253 | Yue et al. 2001[1] |
| SCN 25-2 | Linkage | BARC-027452-06569 | 1788836 | Satt309 | 1736253 | Yue et al. 2001[1] |
| SCN 26-3 | Linkage | BARC-027452-06569 | 1788836 | Satt309 | 1736253 | Yue et al. 2001[1] |
| SCN 27-1 | Linkage | BARC-027452-06569 | 1788836 | Satt309 | 1736253 | Meksem et al. 2001C[13] |
| SCN 32-1 | Linkage | Sat_141 | 2409363 | BARC-035305-07162 | 1726852 | Ferdous et al. 2006[22] |
| SCN 4-4 | Linkage | Sat_141 | 2409363 | BARC-035305-07162 | 1726852 | Concibido et al. 1996[30] |
| SCN 5-3 | Linkage | Sat_141 | 2409363 | BARC-035305-07162 | 1726852 | Concibido et al. 1996[30] |
| SCN 6-2 | Linkage | Sat_141 | 2409363 | BARC-035305-07162 | 1726852 | Concibido et al. 1996[30] |
| SCN 7-1 | Linkage | Sat_141 | 2409363 | BARC-035305-07162 | 1726852 | Concibido et al. 1996[30] |
| SCN 28-1 | Linkage | Sat_141 | 2409363 | Satt309 | 1736253 | Glover et al. 2004[26] |

| | | | | | | |
|---|---|---|---|---|---|---|
| SCN 28-3 | Linkage | Sat_141 | 2409363 | Satt309 | 1736253 | Glover et al. 2004[26] |
| SCN 42-2 | Linkage | Sat_163 | 2410304 | BARC-035305-07162 | 1726852 | Ariagada et al. 2012[8] |
| SCN 14-1 | Linkage | Sat_163 | 2410304 | Satt309 | 1736253 | Meksem et al. 1999[29] |
| SCN 8-2 | Linkage | Sat_163 | 2410304 | Satt309 | 1736253 | Chang et al. 1997[17] |
| SCN 39-7 | Linkage | Satt610 | 2664941 | Satt309 | 1736253 | Wu et al. 2009[5] |
| SCN 8-1 | Linkage | Satt570 | 3162690 | Sat_163 | 2410304 | Chang et al. 1997[17] |
| SCN 33-1 | Linkage | Satt688 | 3264226 | Satt309 | 1736253 | Guo et al. 2006[6] |
| SCN 29-1 | Overlap | Satt688 | 3264226 | Satt163 | 998312 | Guo et al. 2006[23] |
| SCN 29-4 | Overlap | Satt688 | 3264226 | Satt163 | 998312 | Guo et al. 2006[23] |
| SCN 29-8 | Linkage | Satt688 | 3264226 | Satt163 | 998312 | Guo et al. 2006[23] |
| SCN 42-4 | Linkage | Satt217 | 4692233 | Sat_141 | 2409363 | Ariagada et al. 2012[8] |
| SCN 15-1 | Linkage | BARC-041147-07917 | 6085107 | Satt309 | 1736253 | Vaghchhipawala et al. 2001[31] |

1: race 3 equals to Hg type 0; race 4 equals to Hg type 1,2,3,5,7

Reference


1. Yue P, Sleper DA, Arelli PR: **Mapping resistance to multiple races of heterodera glycines in soybean PI 89772**. *Crop Sci* 2001, **41**(5):1589-1595.
2. Yue P, Arelli PR, Sleper DA: **Molecular characterization of resistance to Heterodera glycines in soybean PI 438489B**. *Theor Appl Genet* 2001, **102**(6-7):921-928.
3. Vuong T, Sleper D, Shannon J, Wu X, Nguyen H: **Confirmation of quantitative trait loci for resistance to multiple-HG types of soybean cyst nematode (Heterodera glycines Ichinohe)**. *Euphytica* 2011, **181**(1):101-113.
4. Ferreira MFD, Cervigni GDL, Ferreira A, Schuster I, Santana FA, Pereira WD, de Barros EG, Moreira MA: **QTLs for resistance to soybean cyst nematode, races 3, 9, and 14 in cultivar Hartwig**. *Pesquisa Agropecuaria Brasileira* 2011, **46**(4):420-428.
5. Wu X, Blake S, Sleper D, Shannon JG, Cregan P, Nguyen H: **QTL, additive and epistatic effects for SCN resistance in PI 437654**. *Theor Appl Genet* 2009, **118**(6):1093-1105.

6. Guo B, Sleper DA, Nguyen HT, Arelli PR, Shannon JG: **Quantitative trait loci underlying resistance to three soybean cyst nematode populations in soybean PI 404198A**. *Crop Sci* 2006, **46**(1):224-233.
7. Mahalmgam R, Skorupska HT: **DNA Markers for Resistance to Heterodera glycines I. Race 3 in Soybean Cultivar Peking**. *Japanese Journal of Breeding* 1995, **45**(4):435-443.
8. Arriagada O, Mora F, Dellarossa JC, Ferreira MFS, Cervigni GDL, Schuster I: **Bayesian mapping of quantitative trait loci (QTL) controlling soybean cyst nematode resistant**. *Euphytica* 2012, **186**(3):907-917.
9. Concibido V, Denny R, Boutin S, Hautea R, Orf J, Young N: **DNA marker analysis of loci underlying resistance to soybean cyst nematode (Heterodera glycines Ichinohe)**. *Crop Sci* 1994, **34**(1):240-246.
10. Matthews BF, MacDonald MH, Gebhardt JS, Devine TE: **Molecular markers residing close to the Rhg4 locus conferring resistance to soybean cyst nematode race 3 on linkage group A of soybean**. *Theor Appl Genet* 1998, **97**(7):1047-1052.
11. Prabhu RR, Njiti VN, Bell-Johnson B, Johnson JE, Schmidt ME, Klein JH, Lightfoot DA: **Selecting soybean cultivars for dual resistance to soybean cyst nematode and sudden death syndrome using two DNA markers**. *Crop Sci* 1999, **39**(4):982-987.
12. Meksem K, Ruben E, Hyten D, Schmidt M, Lightfoot D: **High-throughput genotyping for a polymorphism linked to soybean cyst nematode resistance gene Rhg4 by using Taqman (TM) probes**. *Mol Breed* 2001, **7**(1):63-71.
13. Meksem K, Pantazopoulos P, Njiti VN, Hyten LD, Arelli PR, Lightfoot DA: **'Forrest' resistance to the soybean cyst nematode is bigenic: saturation mapping of the Rhg1and Rhg4 loci**. *Theor Appl Genet* 2001, **103**(5):710-717.
14. Yuan J, Njiti VN, Meksem K, Iqbal MJ, Triwitayakorn K, Kassem MA, Davis GT, Schmidt ME, Lightfoot DA: **Quantitative trait loci in Two Soybean Recombinant Inbred Line Populations Segregating for Yield and Disease Resistance**. *Crop Sci* 2002, **42**(1):271-277.
15. Meksem K, Ruben E Fau - Hyten D, Hyten D Fau - Triwitayakorn K, Triwitayakorn K Fau - Lightfoot DA, Lightfoot DA: **Conversion of AFLP bands into high-throughput DNA markers**. *Mol Genet Genomics* 2001, **265**(2):207-214.
16. Webb DM, Baltazar BM, Rao-Arelli AP, Schupp J, Clayton K, Keim P, Beavis WD: **Genetic mapping of soybean cyst nematode race-3 resistance loci in the soybean PI 437.654**. *TAG Theoretical and applied genetics Theoretische und angewandte Genetik* 1995, **91**(4):574-581.
17. Chang SJC, Doubler TW, Kilo VY, Abu-Thredeih J, Prabhu R, Freire V, Suttner R, Klein J, Schmidt ME, Gibson PT *et al*: **Association of loci underlying field resistance to soybean sudden death syndrome (SDS) and cyst nematode (SCN) race 3**. *Crop Sci* 1997, **37**(3):965-971.
18. Kazi S, Shultz J, Afzal J, Hashmi R, Jasim M, Bond J, Arelli PR, Lightfoot DA: **Iso-lines and inbred-lines confirmed loci that underlie resistance from cultivar 'Hartwig' to three soybean cyst nematode populations**. *Theor Appl Genet* 2010, **120**(3):633-644.

19. Brucker E, Carlson S, Wright E, Niblack T, Diers B: **Rhg1 alleles from soybean PI 437654 and PI 88788 respond differentially to isolates of Heterodera glycines in the greenhouse**. *Theor Appl Genet* 2005, **111**(1):44-49.
20. Vuong T, Sleper D, Shannon J, Nguyen H: **Novel quantitative trait loci for broad-based resistance to soybean cyst nematode (Heterodera glycines Ichinohe) in soybean PI 567516C**. *Theor Appl Genet* 2010, **121**(7):1253-1266.
21. Vierling RA, Faghihi J, Ferris VR, Ferris JM: **Association of RFLP markers with loci conferring broad-based resistance to the soybean cyst nematode (Heterodera glycines)**. *TAG Theoretical and applied genetics Theoretische und angewandte Genetik* 1996, **92**(1):83-86.
22. Ferdous SA, Watanabe S, Suzuki-Orihara C, Tanaka Y, Kamiya M, Yamanaka N, Harada K: **QTL analysis of resistance to soybean cyst nematode race 3 in soybean cultivar toyomusume**. *Breeding Science* 2006, **56**(2):155-163.
23. Guo B, Sleper DA, Arelli PR, Shannon JG, Nguyen HT: **Identification of QTLs associated with resistance to soybean cyst nematode races 2, 3 and 5 in soybean PI 90763 (vol 111, pg 965, 2005)**. *Theor Appl Genet* 2006, **112**(5):984-985.
24. Qiu BX, Arelli PR, Sleper DA: **RFLP markers associated with soybean cyst nematode resistance and seed composition in a ‘Peking’×‘Essex’ population**. *Theor Appl Genet* 1999, **98**(3-4):356-364.
25. Chang W, Dong L, Wang Z, Hu H, Han Y, Teng W, Zhang H, Guo M, Li W: **QTL underlying resistance to two HG types of Heterodera glycines found in soybean cultivar 'L-10'**. *BMC Genomics* 2011, **12**:233.
26. Glover KD, Wang D, Arelli PR, Carlson SR, Cianzio SR, Diers BW: **Near isogenic lines confirm a soybean cyst nematode resistance gene from PI 88788 on linkage group J**. *Crop Sci* 2004, **44**(3):936-941.
27. Concibido V, Lange D, Denny R, Orf J, Young N: **Genome mapping of soybean cyst nematode resistance genes in Peking, PI90763, and PI88788 using DNA markers**. *Crop Sci* 1997, **37**(1):258-264.
28. Mudge J, Cregan PB, Kenworthy JP, Kenworthy WJ, Orf JH, Young ND: **Two microsatellite markers that flank the major soybean cyst nematode resistance locus**. *Crop Sci* 1997, **37**(5):1611-1615.
29. Meksem K, Doubler TW, Chancharoenchai K, Nijti N, Chang SJ, Arelli APR, Cregan PE, Gray LE, Gibson PT, Lightfoot DA: **Clustering among loci underlying soybean resistance to Fusarium solani, SDS and SCN in near-isogenic lines**. *Theor Appl Genet* 1999, **99**(7-8):1131-1142.
30. Concibido VC, Young ND, Lange DA, Denny RL, Danesh D, Orf JH: **Targeted comparative genome analysis and qualitative mapping of a major partial-resistance gene to the soybean cyst nematode**. *Theor Appl Genet* 1996, **93**(1-2):234-241.
31. Vaghchhipawala Z, Bassuner R, Clayton K, Lewers K, Shoemaker R, Mackenzie S: **Modulations in gene expression and mapping of genes associated with cyst nematode infection of soybean**. *Molecular plant-microbe interactions : MPMI* 2001, **14**(1):42-54.
